# Supplementary material for: Predicting visual function by interpreting a neuronal wiring diagram
Source: Nature. 2024 Oct 2;634(8032):113–23. doi: 10.1038/s41586-024-07953-5 (PMC11446822; doi:10.1038/s41586-024-07953-5)
Supplement: Supplementary file 6 — CRF and ERF predictions for individual TmY4 and TmY9 cells. Analogous to Supplementary Data 3, but for TmY target types. Shown are the top four monosynaptic pathways, the strongest pathway passing through each of the top ten intermediary types (ranking from Extended Data Fig. 7), and the trisynaptic pathway Tm1–TmY–Dm3–TmY (see the section entitled Prediction of spatial normalization). [file 41586_2024_7953_MOESM6_ESM.zip › DataS4/TmY9qΓèÑ/720575940618241819.pdf]

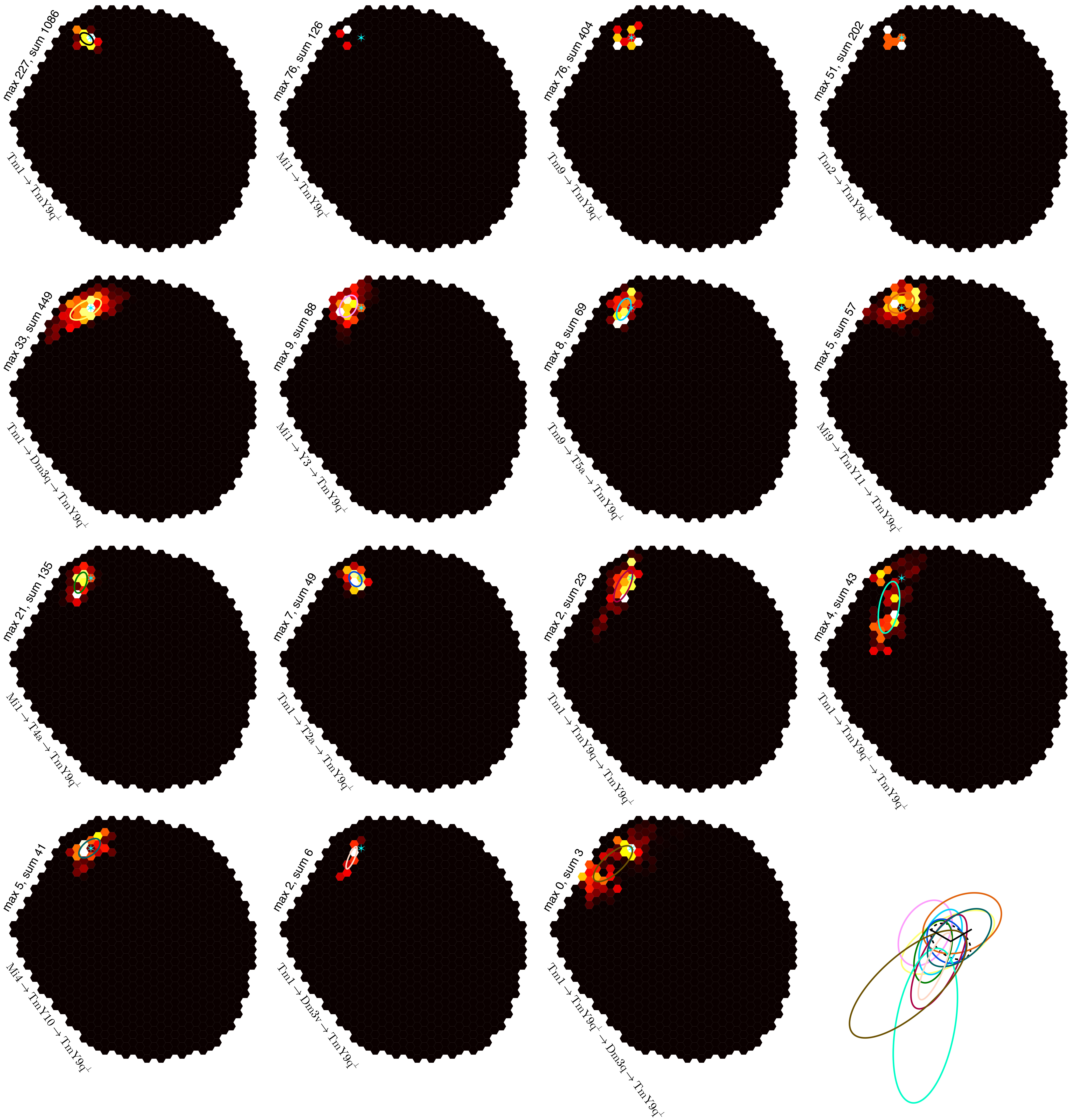
max 227, sum 1086
Tm1 → TmY9q⊥
max 76, sum 126
Mi1 → TmY9q⊥
max 76, sum 404
Tm9 → TmY9q⊥
max 51, sum 202
Tm2 → TmY9q⊥
max 33, sum 449
Tm1 → Dm3q → TmY9q⊥
max 9, sum 88
Mi1 → Y3 → TmY9q⊥
max 8, sum 69
Tm9 → T5a → TmY9q⊥
max 5, sum 57
Mi9 → TmY11 → TmY9q⊥
max 21, sum 135
Mi1 → T4a → TmY9q⊥
max 7, sum 49
Tm1 → T2a → TmY9q⊥
max 2, sum 23
Tm1 → TmY9q → TmY9q⊥
max 4, sum 43
Tm1 → TmY9q⊥ → TmY9q⊥
max 5, sum 41
Mi4 → TmY10 → TmY9q⊥
max 2, sum 6
Tm1 → Dm3v → TmY9q⊥
max 0, sum 3
Tm1 → TmY9q⊥ → Dm3q → TmY9q⊥
